# HIV protease: late action to prevent immune detection

**DOI:** 10.1038/s41392-021-00588-2

**Published:** 2021-04-16

**Authors:** Konstantin M. J. Sparrer, Frank Kirchhoff

**Affiliations:** Institute of Molecular Virology, Ulm University Medical Center, Ulm, Germany

**Keywords:** Immunological disorders, Inflammation

**Fig. 1:**
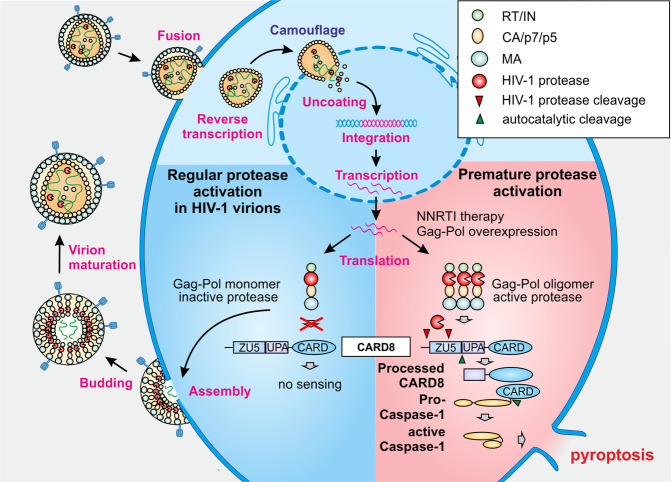
Activation of the CARD8 inflammasome by premature, intracellular activation of the HIV-1 protease. During regular HIV-1 infection incoming capsids are uncoated at the nuclear pore or even in the nucleus, allowing the delivery of the reverse-transcribed linear dsDNA for proviral integration into the host genome. Subsequent transcription and translation lead to expression of the Gag-Pol precursor polyprotein. Assembly and oligomerization of the polyprotein in the budding virion activates the protease, which processes and matures the virion. During NNRTI therapy or artificial Gag-Pol expression, the polyprotein accumulates in the cytoplasm leading to premature viral protease activity. Subsequent cleavage of CARD8 leads to the recruitment of pro-caspase-1, generation of active caspase-1, and ultimately pyroptotic death of HIV-1-infected cells. RT reverse transcriptase, IN integrase, CA capsid protein, MA matrix protein

A recent study published in *Science*^1^ by Wang and colleagues shows that premature activation of the HIV-1 protease leads to caspase activation and recruitment domain 8 (CARD8) inflammasome-mediated pyroptosis of infected macrophages and CD4+ T cells (Fig. 1). These findings might help to improve approaches aiming to eliminate the latent viral reservoirs upon HIV-1 reactivation.

Most invading viral pathogens trigger the innate immune system and are usually rapidly controlled and eventually eliminated. Cells sense viral intruders via so-called pattern recognition receptors (PRRs) capable of detecting pathogen-associated molecular patterns (PAMPs) allowing discrimination between self and non-self. In many cases, these patterns are viral nucleic acids that show pathogen-specific structures or emerge at the wrong localization in the cell. Ultimately, activation of PRRs by their respective ligands leads to the induction of various anti-viral signaling cascades, among them the inflammasome.

HIV-1, the main causative agent of AIDS, however, is well known for its ability to evade innate immune sensing. For example, accumulating evidence shows that the viral capsid remains largely intact upon viral entry thereby shielding the intermediates of the reverse transcription (RT) process against pattern recognition.^2^ The integrated provirus does not have unusual features and is thus invisible to the immune system. Thus, in the absence of antiviral therapy, efficient immune evasion allows HIV-1 to maintain high levels of replication over several years until the immune system is exhausted and infected individuals develop AIDS.

HIV-1 proteins do usually not act as PAMPs, although it is under debate whether the viral capsid protein might be sensed to stimulate significant innate immune activation in infected cells.^3^ Wang and co-workers now demonstrate that premature activation of the viral protease stimulates the CARD8 inflammasome and induces pyroptosis of HIV-1-infected CD4+ T cells. Their results identify a novel mechanism allowing innate immune sensing of HIV-1 and expand previous data showing that CARD8 inflammasome activation triggers pyroptosis in CD4+ T cells.^4^ Usually, the HIV-1 protease is kept in an inactive state in infected cells and is only liberated from the Gag-Pol polyprotein precursor by autoprocessing, requiring transient dimer formation in the maturing virion. However, the protease can be prematurely activated e.g. by intracellular overexpression of the Gag-Pol precursor or treatment with certain non-nucleosidic reverse transcriptase inhibitors (NNRTIs). Wang et al. show that active intracellular HIV-1 protease cleaves CARD8 twice at its N-terminus, thereby facilitating its autoproteolytic processing and assembly of CARD8 inflammasomes, promoting recruitment and activation of caspase-1. Subsequently, caspase-1 mediates proteolytic cleavage, maturation and release of the pro-inflammatory cytokines interleukin 1β (IL-1β) and IL-18. In addition, caspase-1 leads to cleavage of the pore-forming cell death executor Gasdermin-D (GSDMD), which in turn ruptures the cytoplasmic membrane and induces a programmed, but highly inflammatory form of cell death called pyroptosis (Fig. 1). This novel mechanism adds to the evidence that inflammasome sensors may be triggered by proteins of pathogens. For example, it is known that the NAIP/NLR4C inflammasome can be activated by bacterial flagellins or the bacterial type-3 secretion system. In addition, induction of the mouse NLRP1b inflammasome by the *Bacillus anthracis* lethal factor involves proteolytic activation of this sensor.

CARD8 is efficiently expressed in CD4+ T cells and macrophages representing the major target cells of HIV-1 in infected individuals. The authors show that NNRTI-induced premature activity of the HIV-1 protease triggered CARD8-dependent pyroptosis in macrophages and CD4+ T cells. This effect could be blocked by caspase-1 inhibition, proteasome inhibition, or deletion of CARD8. Notably, treatment with NNRTIs to prematurely activate the HIV-1 protease, also triggered cell death of patient-derived latently infected cells after viral reactivation. Thus, the authors propose that this strategy may help to target the latent reservoirs in the so-called “shock and kill” approach. The ultimate aim of this strategy is to cure HIV-1 by treatment with drugs that activate latent proviruses to induce viral gene expression (shock) thereby rendering existing HIV-1 infected cells vulnerable to elimination (kill), while preventing de novo infection by effective antiretroviral therapy (ART). However, the concentrations of NNRTIs required to trigger pyroptotic cell death are relatively high and it remains to be determined whether this approach might help to eliminate latently infected T cells in patients. It is important to note, that the shock-and-kill strategy is still a largely theoretical concept and thus far evidence that it might achieve a significant reduction of viral reservoirs and a real clinical benefit is lacking. Induction of pyroptotic cell death may even have detrimental effects since the release of pro-inflammatory cytokines and damage-associated molecular patterns upon cell-membrane rupturing may promote harmful chronic inflammation known to drive progression to AIDS as well as accelerated aging and neurological symptoms.

It has been previously suggested that untimely uncoating of the HIV-1 capsid is associated with immune activation because the viral nucleic acids become exposed to cytoplasmic DNA sensors, such as cGAS. In addition, it has been reported that abortive HIV-1 infection induces caspase-1-mediated pyroptosis of quiescent lymphoid CD4 T-cells because reverse transcription may be stalled, giving rise to incomplete cytosolic viral DNA transcripts.^5^ Active protease contained in incoming HIV-1 virions should also be able to trigger CARD8-dependent inflammasome activation. Thus, if the cone-shaped capsid is prematurely disrupted and the virion-associated enzymes released into the cytoplasm CARD8-dependent pyroptosis may be triggered. It will be important to further dissect the contribution of these different mechanisms to HIV-1 innate immune sensing and cell death. Finally, polyprotein processing by viral proteases is a strategy used by many viral pathogens. Consequently, it will be interesting to determine whether proteases of other viruses are also able to activate the CARD8 inflammasome.
